# Nuclear localization is required for Dishevelled function in Wnt/β-catenin signaling

**DOI:** 10.1186/jbiol20

**Published:** 2005-02-15

**Authors:** Keiji Itoh, Barbara K Brott, Gyu-Un Bae, Marianne J Ratcliffe, Sergei Y Sokol

**Affiliations:** 1 Department of Microbiology and Molecular Genetics, Harvard Medical School, and Beth Israel Deaconess Medical Center, Boston, MA 02215, USA; 2 Current address: Department of Molecular, Cell and Developmental Biology, Mount Sinai School of Medicine, Box 1020, One Gustave L. Levy Place, New York, NY 10029, USA

## Abstract

**Background:**

Dishevelled (Dsh) is a key component of multiple signaling pathways that are initiated by Wnt secreted ligands and Frizzled receptors during embryonic development. Although Dsh has been detected in a number of cellular compartments, the importance of its subcellular distribution for signaling remains to be determined.

**Results:**

We report that Dsh protein accumulates in cell nuclei when *Xenopus *embryonic explants or mammalian cells are incubated with inhibitors of nuclear export or when a specific nuclear-export signal (NES) in Dsh is disrupted by mutagenesis. Dsh protein with a mutated NES, while predominantly nuclear, remains fully active in its ability to stimulate canonical Wnt signaling. Conversely, point mutations in conserved amino-acid residues that are essential for the nuclear localization of Dsh impair the ability of Dsh to activate downstream targets of Wnt signaling. When these conserved residues of Dsh are replaced with an unrelated SV40 nuclear localization signal, full Dsh activity is restored. Consistent with a signaling function for Dsh in the nucleus, treatment of cultured mammalian cells with medium containing Wnt3a results in nuclear accumulation of endogenous Dsh protein.

**Conclusions:**

These findings suggest that nuclear localization of Dsh is required for its function in the canonical Wnt/β-catenin signaling pathway. We discuss the relevance of these findings to existing models of Wnt signal transduction to the nucleus.

## Background

The specification of cell fates during embryonic development frequently depends on inductive interactions, which involve transmission of extracellular signals from the cell surface to the nucleus. In the transforming growth factor β (TGFβ) signal transduction pathway, Smad proteins that are initially associated with TGFβ receptors move to the nucleus to regulate target genes [1]. Another example of a direct link between the cell surface and the nucleus during embryonic development is the proteolytic cleavage and nuclear translocation of the cytoplasmic fragment of the Notch receptor [2]. In contrast, multiple steps appear to be required for a Wnt signal to reach the nucleus. In this molecular pathway, signals from Frizzled receptors are transduced to Dishevelled (Dsh), followed by inactivation of the β-catenin degradation complex that includes the adenomatous polyposis coli protein (APC), Axin and glycogen synthase kinase 3 (GSK3) [3,4]. Stabilization of β-catenin is thought to promote its association with members of the T-cell factor (Tcf) transcription factor family in the nucleus, resulting in the activation of target genes [5,6]. As well as the canonical β-catenin-dependent pathway, Frizzled receptors also activate small GTPases of the Rho family, protein kinase C and Jun-N-terminal kinases (JNKs) to regulate planar cell polarity in *Drosophila *and convergent extension cell movements and tissue separation in *Xenopus *[7-12]. Thus, the Wnt/Frizzled pathway serves as a model for molecular target selection during signal transduction.

Dsh is a common intracellular mediator of several pathways activated by Frizzled receptors and is composed of three conserved regions that are known as the DIX, PDZ and DEP domains [13]. Different domains of Dsh are engaged in specific interactions with different proteins, thereby leading to distinct signaling outcomes [13]. Daam, a formin-related protein, promotes RhoA activation by Dsh [9], whereas Frodo, another Dsh-binding protein, is required for Wnt/β-catenin signaling in the nucleus [14]. These interactions may take place in various cellular compartments, linking specific activities of Dsh to its distribution inside the cell. Dsh is found in a complex with microtubules and with the actin cytoskeleton [15-17]. Dsh is also associated with cytoplasmic lipid vesicles, and this localization was shown to require the DIX domain [7,16,18]. Overexpressed Frizzled receptors can recruit Dsh to the cell membrane in *Xenopus *ectoderm, and this redistribution requires the DEP domain [7,18,19]. The DIX domain is essential for the Wnt/β-catenin pathway, whereas the DEP domain plays a role in the planar cell polarity pathway [7,8,16,18,20,21]. Thus, the specific subcellular localization of Dsh may be crucial for local signaling events.

The current study was based on our initial observation that a Dsh construct lacking the carboxy-terminal DEP domain was found in cell nuclei. We have now identified a nuclear export signal in the deleted region and also discovered that Dsh proteins accumulate in the nuclei of *Xenopus *ectodermal cells and mammalian cells upon inhibition of nuclear export. Dsh also accumulated in the nuclei after stimulation of mammalian cells with Wnt3a-containing culture medium. By analyzing various mutant Dsh constructs in *Xenopus *ectoderm, we show that the signals responsible for Dsh nuclear localization reside in a novel domain and that the nuclear translocation of Dsh is essential for its ability to activate Wnt/β-catenin signaling.

## Results and discussion

### A nuclear export signal in Dsh is responsible for the cytoplasmic localization of Dsh

**Figure 1:**
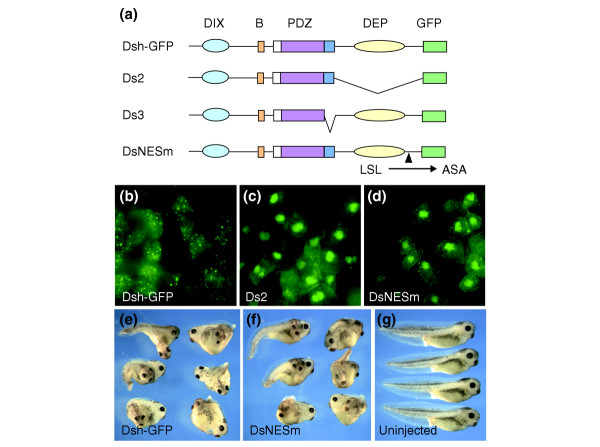
Nuclear export of Dsh is not critical for its activity. **(a) **The Dsh constructs used to analyze nuclear export. **(b-d) **RNAs encoding Dsh-GFP, Ds2 and DsNESm (0.5 ng each) were injected into two animal blastomeres of 4–8-cell embryos. Animal-cap explants were excised at stage 10, fixed and examined for GFP fluorescence. (b) Wild type Dsh-GFP localized in punctate structures of the cytoplasm, whereas (c) Ds2 and (d) DsNESm accumulated in the nucleus of animal pole cells. **(e,f) **One ventral vegetal blastomere of 8-cell embryos was injected with 1 ng Dsh-GFP or DsNESm RNA as indicated. Complete secondary axes were induced in both cases. **(g) **Uninjected sibling embryos.

We studied the subcellular distribution of fusions of Dsh with green fluorescent protein (GFP) in *Xenopus *ectodermal cells. In contrast to Dsh-GFP, which is localized in punctate structures within the cytoplasm [7,18], the Ds2 construct, lacking the carboxy-terminal region, accumulates in the nucleus (Figure 1a–c), indicating that the carboxyl terminus contains sequences for nuclear export. Indeed, we found a potential leucine-rich nuclear export signal (NES) in Dsh at positions 510–515, corresponding to the conserved consensus M/LxxLxL (single letter amino-acid code, where x is any amino acid) [22,23]. When leucines 513 and 515 in this putative NES were substituted with alanines, the mutated Dsh fusion construct, DsNESm, was localized predominantly in the nucleus (Figure 1a,d), demonstrating that the sequence is a functional nuclear export signal.

To examine whether inhibition of nuclear export abrogates Dsh activity, we compared the abilities of DsNESm and wild-type Dsh-GFP to induce secondary axes in frog embryos. Although the molecular mechanism operating during axis induction remains to be elucidated, this assay faithfully reflects the biological activity of Dsh in the canonical Wnt/β-catenin pathway [14,16,18,24]. DsNESm, which was expressed at similar levels to the wild-type Dsh-GFP (data not shown), induced secondary axes at least as efficiently as Dsh-GFP (Table 1). Induced axes contained pronounced head structures with eyes and cement glands (Figure 1e–g). These results suggest that Dsh may function in the nucleus to trigger dorsal axial development.

### Nuclear localization of Dsh in cells treated with nuclear export inhibitors

**Figure 2:**
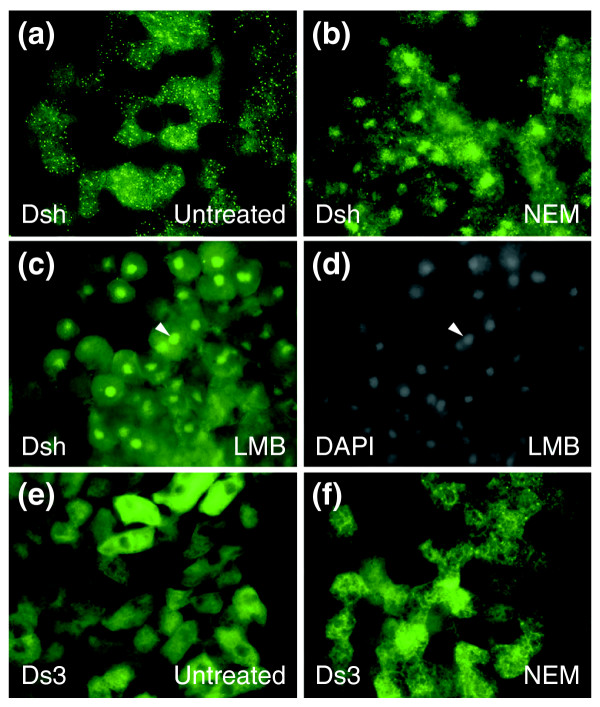
Accumulation of Dsh in the nucleus in the absence of nuclear export. **(a-d) **Dsh-GFP RNA (0.7 ng) was injected into two animal blastomeres of 4–8 cell embryos. Animal caps were excised at stage 10 and then left (a) untreated or (b) treated with 10 mM NEM or (c,d) 50 ng/ml leptomycin B (LMB), fixed and examined for GFP fluorescence. (a) Dsh-GFP is mainly localized to vesicular structures in the cytoplasm. In the presence of (b) NEM or (c) LMB, Dsh-GFP accumulates in the nucleus, as supported by (d) DAPI staining of nuclei in the same field as in (c). Nuclear staining is marked by arrowheads (c,d). **(e,f) **The Ds3 construct, lacking amino acids 334–381, remained in the cytoplasm in the (e) absence or (f) presence of NEM.

Accumulation of DsNESm in the nucleus implies that the wild-type Dsh shuttles between the nucleus and the cytoplasm. We therefore studied the subcellular distribution of Dsh in *Xenopus *embryonic cells under conditions in which nuclear export is blocked. When ectodermal cells expressing Dsh-GFP were incubated with N-ethylmaleimide (NEM), an inhibitor of the nuclear export receptor CRM1/exportin [25,26], Dsh-GFP was detected predominantly in the nucleus, compared to the punctate cytoplasmic pattern of Dsh-GFP in untreated cells (Figure 2a,b). This effect was specific to full-length Dsh-GFP, as Ds3, a Dsh construct that lacks 48 amino acids adjacent to the PDZ domain (Figure 1a), did not accumulate in the nucleus after NEM treatment (Figure 2e,f). The nuclear retention of Dsh-GFP was also observed using leptomycin B (LMB), another inhibitor of CRM1-dependent nuclear export [22,23] (Figure 2c,d). These results indicate that Dsh shuttles between the cytoplasm and the nucleus, and that its abundance in the cytoplasm is due to highly efficient nuclear export.

**Figure 3:**
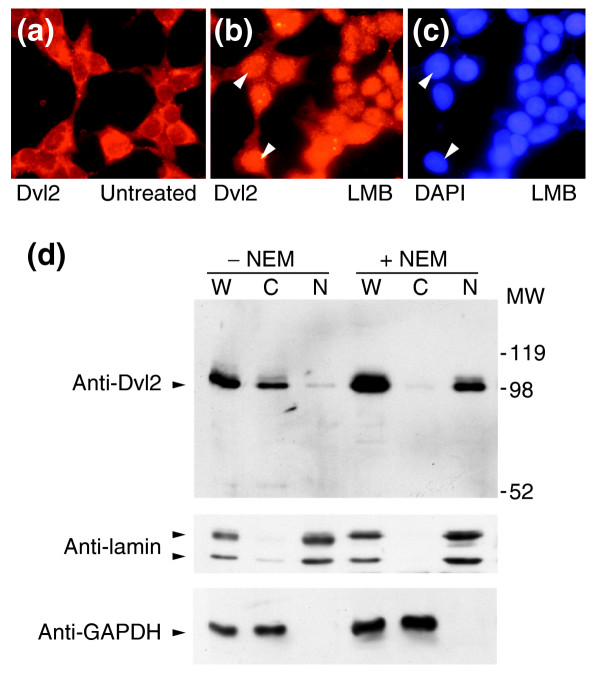
Endogenous Dsh shuttles between the cytoplasm and nucleus. Immunofluorescent staining of HEK293 cells with anti-Dvl2 antibodies reveals different subcellular localization of Dvl2 **(a) **without or **(b) **with LMB treatment. **(c) **DAPI staining shows the location of nuclei in the same field as (b); the arrowheads indicate corresponding nuclei in (b) and (c). **(d) **Distribution of endogenous Dvl2 recognized by anti-Dvl2 antibodies in the nuclear and the cytoplasmic fractions of Rat-1 fibroblasts. In the absence of NEM, Dvl2 is localized mainly in the cytoplasm (C), while after NEM treatment Dvl2 is exclusively localized in the nuclei (N). W, whole cell lysate. Antibodies to lamin and GAPDH show the separation of the nuclear and cytoplasmic fractions.

To ensure that the Dsh-GFP fusion behaves similarly to the endogenous Dsh protein, we examined the localization of endogenous Dvl2, a mammalian homolog of Dsh, in human and rat tissue culture cells. Human embryonic kidney (HEK) 293 cells treated with LMB accumulated Dvl2 in the nucleus, contrasting with the cytoplasmic localization of Dvl2 in untreated cells (Figure 3a–c). We also evaluated the subcellular localization of endogenous Dvl2 in Rat-1 fibroblasts, which are known to respond to Wnt signaling. Fractionation of cells into nuclear and cytoplasmic protein pools revealed only a small amount of endogenous Dvl2 in intact nuclei, whereas after NEM treatment, Dvl2 was localized predominantly in the nuclear fraction (Figure 3d). The efficiency of subcellular fractionation was controlled for by staining with antibodies to glyceraldehyde phosphate dehydrogenase (GAPDH) and nuclear lamins. These proteins remained exclusively cytoplasmic or nuclear, respectively, in both untreated and NEM-treated cells (Figure 3d). Thus, our data show that Dsh translocates into the nucleus and is actively exported into the cytoplasm of both *Xenopus *ectodermal cells and mammalian fibroblasts.

### Identification of sequences responsible for Dsh nuclear localization

**Figure 4:**
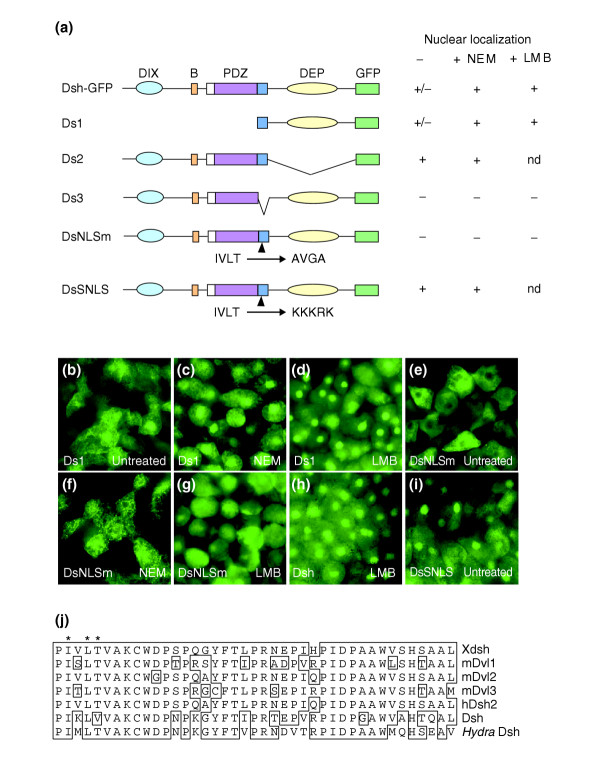
Mapping nuclear localization signals in Dsh. **(a) **The Dsh constructs used to study nuclear transport and their localization to the nucleus after NEM or LMB treatment; the DIX, PDZ and DEP domains are shown as in Figure 1a; B is the basic region and nd denotes not done. **(b-i) **Subcellular localization of Dsh-GFP constructs in the absence or presence of NEM or LMB. Embryos were injected with 0.5 ng of each mRNA, and GFP analysis was carried out as in Figure 1b-d. (b-d) Ds1, (e-g) DsNLSm, (h) Dsh, (i) DsSNLS. (b,e,i) no NEM treatment; (c,f) after NEM treatment; (d,g,h) after LMB treatment. **(j) **Comparison of conserved amino-acid sequences that are required for Dsh nuclear localization; X denotes the *Xenopus *protein, m the mouse and h the human. Amino-acid residues mutated in DsNLSm are indicated by asterisks.

To identify specific amino-acid sequences that direct the transport of Dsh to the nucleus, we studied the subcellular distribution of mutated Dsh-GFP fusion constructs (Figure 4a). The removal of the DIX and PDZ domains (Ds1) did not eliminate nuclear translocation in response to NEM or LMB (Figure 4a–d), indicating that these two domains are not required for the nuclear import. Similarly, the DEP domain is not required for Dsh nuclear localization (Ds2; Figure 1a,c). Comparison of Ds1 and Ds2 (see Figure 4a), both capable of nuclear localization, reveals a short stretch of shared amino acids located between the PDZ and DEP domains. Strikingly, the removal of just this 48 amino-acid region abrogated nuclear import of Dsh in the presence of NEM or LMB (Ds3; Figures 2e,f and 4a). Together these experiments identify amino acids 333–381 as the region required for nuclear localization of Dsh.

Although this short sequence is highly conserved in all Dsh homologs from *Hydra *to humans (Figure 4j), it does not bear detectable similarity to nuclear localization signals characterized in other proteins [27]. This sequence may interact directly with components of the nuclear import machinery or bind to a protein that itself binds a karyopherin/importin and mediates the nuclear import of Dsh by a piggyback mechanism. Interestingly, this region overlaps a novel proline-rich domain identified by mutational analysis of Dsh in *Drosophila *[28]. To define further the specific amino acids necessary for nuclear localization, a panel of Dsh constructs with point mutations spanning the conserved region was examined (data not shown). Nuclear import was eliminated with the substitution of three amino acids, converting IVLT into AVGA (DsNLSm; Figure 4a,e–g,j), indicating that these three amino acids are critical.

### Dsh nuclear translocation is crucial for its function in the β-catenin pathway

**Figure 5:**
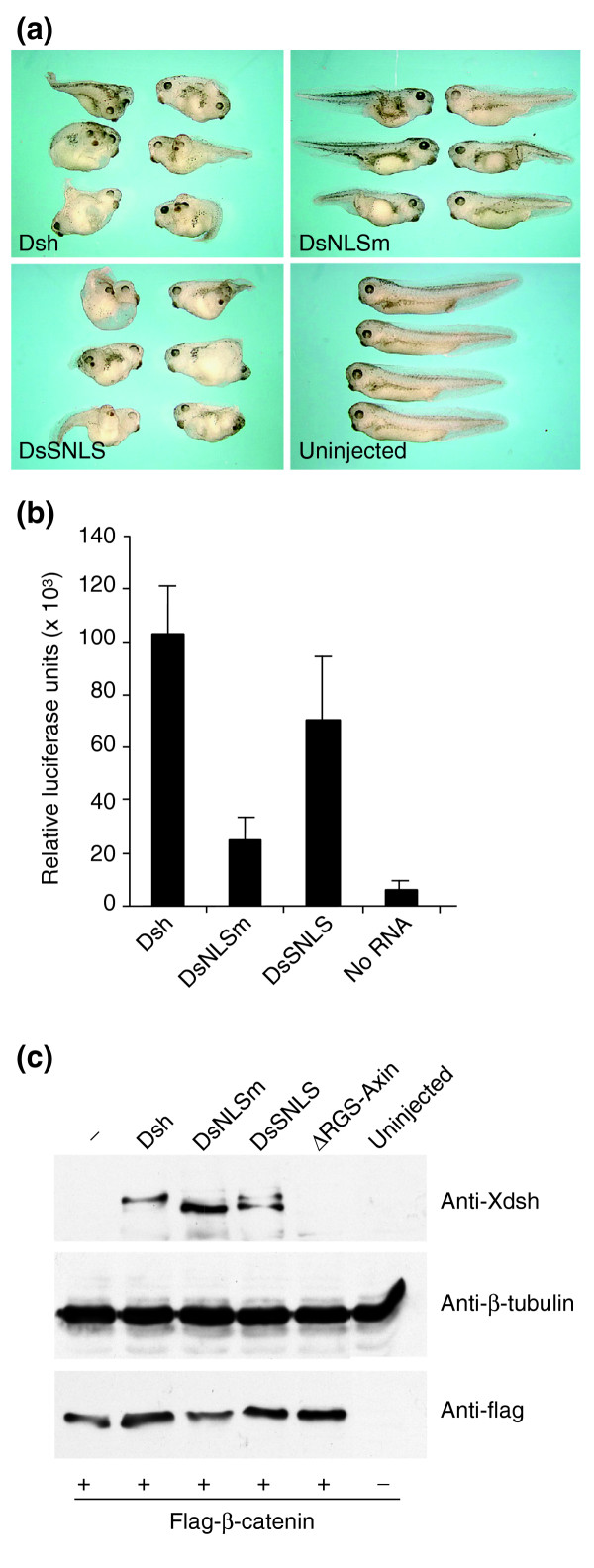
Activation of the Wnt/β-catenin pathway requires nuclear localization of Dsh. **(a) **Axis-inducing activity of Dsh constructs. One ventral vegetal blastomere of 8-cell embryos was injected with 1 ng Dsh-GFP, DsNLSm, or DsSNLS mRNA as indicated. Uninjected sibling embryos are also shown. **(b) **Activation of the *Siamois *reporter gene. The reporter -833pSia-Luc plasmid (20 pg) was coinjected with Dsh-GFP, DsNLSm or DsSNLS mRNA (0.5 ng each) into a single animal ventral blastomere of 8-cell embryos. Injected embryos were lysed at stage 10+ for luciferase activity determination. Results are shown in relative light units as the mean +/- standard deviation from triplicate samples. **(c) **Requirement for Dsh NLS for the stabilization of β-catenin. Flag-β-catenin mRNA (0.4 ng) was coinjected with Dsh, DsNLSm, DsSNLS or ΔRGS-Axin mRNA (2 ng each) into four animal blastomeres of 4–8-cell embryos. Levels of β-catenin and Dsh constructs were assessed in stage 10 embryo lysates with anti-Flag antibodies and anti-Xdsh antibodies; β-tubulin serves as a loading control. Dsh and DsSNLS, but not DsNLSm, are able to stabilize β-catenin. ΔRGS-Axin was used as a control for an activator of the Wnt pathway.

To determine whether nuclear localization of Dsh is required for its activity, we compared the abilities of DsNLSm and wild-type Dsh to induce secondary axes in frog embryos. We also assessed activation of a luciferase reporter construct for *Siamois *[29], an immediate target of Wnt/β-catenin signaling. DsNLSm had impaired ability to induce secondary axes and to activate the *Siamois *reporter when compared with wild-type Dsh (Figure 5a,b; Table 1). Furthermore, DsNLSm failed to stabilize β-catenin (Figure 5c). This difference was not due to differences in protein expression, as both constructs were present in embryo lysates at similar levels (Figure 5c). Thus, these findings indicate that the nuclear localization of Dsh is critical for its functional activity in the β-catenin pathway.

**Figure 6:**
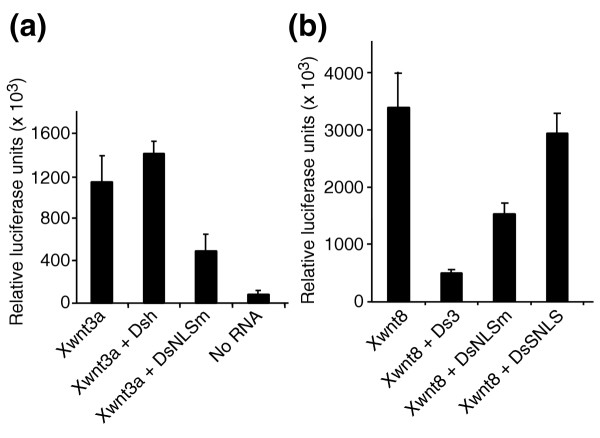
Dominant inhibition of Wnt-dependent transcription by Dsh mutants. Eight-cell embryos were injected **(a) **in one animal ventral blastomere or **(b) **in one vegetal ventral blastomere with -833pSia-Luc DNA (20 pg), mRNAs encoding Xwnt3a (5 pg) or Xwnt8 (2 pg), and Dsh-GFP, DsNLSm, Ds3 or DsSNLS mRNA (0.5 ng) as indicated. Luciferase activity was measured as described in Figure 5b.

Not only was the function of DsNLSm in the β-catenin pathway impaired, but we found that this construct behaved as a dominant inhibitor of Wnt signaling and prevented the activation of the *Siamois *reporter by Xwnt3a and Xwnt8 RNAs (Figure 6a,b). Consistent with these observations, another construct lacking the region responsible for the nuclear localization (Ds3; see Figure 4a) also suppressed Wnt signaling (Figure 6b). Despite these inhibitory properties, dorsally injected DsNLSm RNA, like Xdd1, a dominant negative deletion mutant [24], did not suppress primary axis formation (data not shown).

Impaired activity of the DsNLSm construct may be due to its inability to translocate to the nucleus, or due to a coincidental elimination of a binding site for an essential cofactor that functions together with Dsh in the cytoplasm. To exclude the latter possibility, the IVLT sequence of Dsh NLS was replaced with KKKRK, an unrelated NLS from SV40 T antigen [27]. This construct, DsSNLS, relocated to the nucleus even in the absence of nuclear export inhibitors (Figure 4a,i). Notably, all activities of wild-type Dsh, including induction of complete secondary axes, activation of the *Siamois *promoter and β-catenin stabilization were significantly restored in DsSNLS (Figure 5a–c; Table 1). In contrast to DsNLSm, DsSNLS did not inhibit the ability of Wnt ligands to activate pSia-Luc (Figure 6b), consistent with its being a positive regulator of the Wnt pathway. We note that the signaling activity of DsSNLS was not enhanced compared to wild-type Dsh, suggesting that the rate of the nuclear translocation of Dsh rather than its steady state levels in the nucleus is critical for target gene activation. It is also possible that other nuclear components, rather than Dsh, become rate-limiting for signaling. Overall, the simplest interpretation of our data is that the nuclear import of Dsh is essential for its activity.

**Figure 7:**
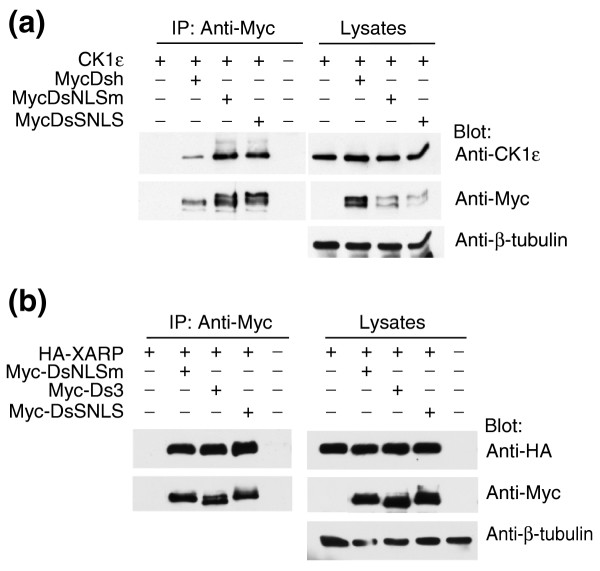
Dsh mutants retain the ability to bind CK1ε and XARP. Four-cell embryos were injected in four sites in the animal hemisphere with CK1ε, HA-XARP, Myc-tagged Dsh, DsNLSm, Ds3 or DsSNLS RNA alone (2 ng each) or in combinations as indicated. The embryonic lysates were collected at stage 10.5 for immunoprecipitation with anti-Myc antibodies. Co-immunoprecipitated **(a) **CK1ε or **(b) **HA-XARP was probed with anti-CK1ε or anti-HA antibodies; β-tubulin served as a loading control.

We next examined the ability of DsNLSm to bind critical Wnt signaling components, such as casein kinase 1ε (CK1ε), a positive regulator of the β-catenin pathway [30,31], or Axin, a negative regulator [20,32-36], both of which are known to bind Dsh. Both DsSNLS, enriched in the nucleus, and DsNLSm and Ds3, which do not enter the nucleus, bound CK1ε and XARP, a *Xenopus *Axin-related protein [20] (Figure 7). Thus, these mutated Dsh constructs retain the ability to associate with critical components of the Wnt/β-catenin pathway, arguing that defective nuclear translocation of DsNLSm is likely to be responsible for its inability to activate β-catenin signaling.

### Suppression of Dsh nuclear import does not affect noncanonical signaling

**Figure 8:**
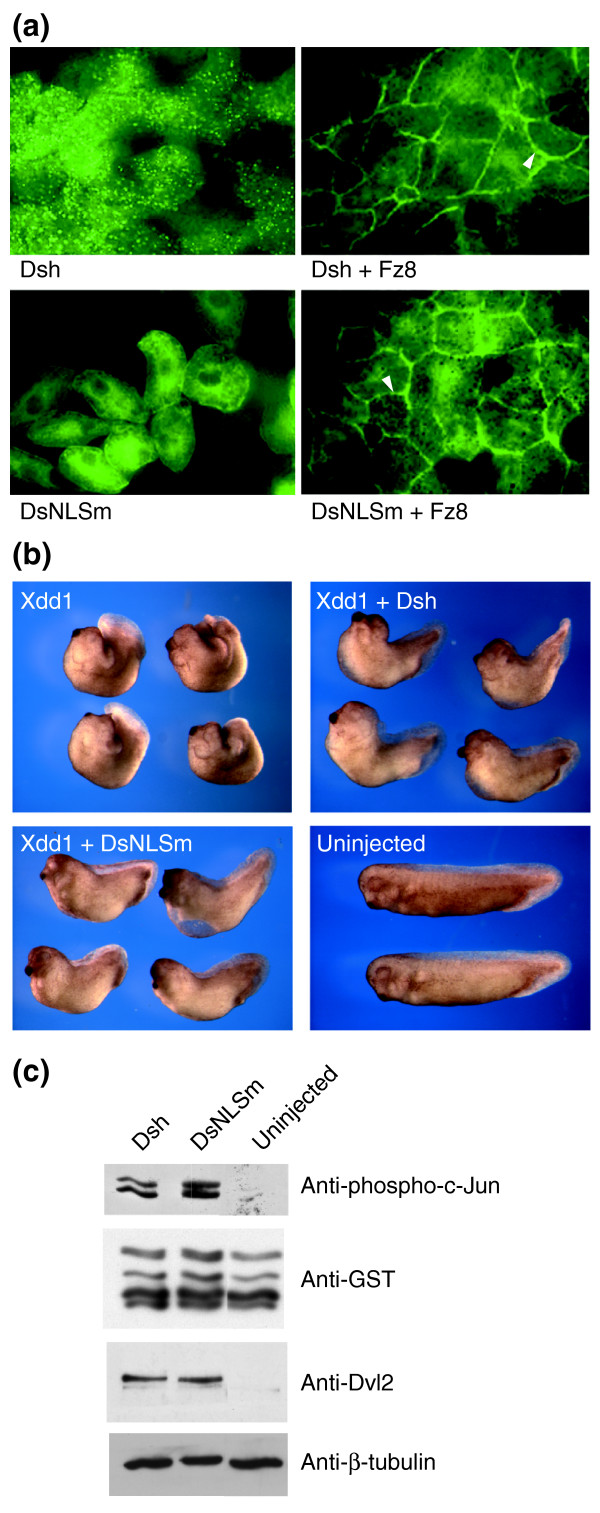
DsNLSm, defective in the β-catenin pathway, is active in noncanonical signaling. **(a) **Fz8-dependent recruitment of Dsh-GFP constructs to the cell membrane. Dsh-GFP or DsNLSm RNA (0.5 ng) was injected alone or with Fz8 RNA (1 ng) into two animal blastomeres at the 4–8-cell stage. GFP fluorescence was assessed in animal cap explants as in Figure 1b-d. Both Dsh and DsNLSm are efficiently recruited to the cell membrane by Fz8. Arrowheads point to cell membranes. **(b) **DsNLSm can rescue convergent extension defects caused by Xdd1. Four-cell embryos were injected with 0.6 ng Xdd1 RNA alone or together with 2 ng Dsh-GFP or DsNLSm RNA into two vegetal dorsal blastomeres. The injected embryos were allowed to develop until the sibling embryos reached stage 32. **(c) **Activation of JNK by the Dsh nuclear import mutant. Four animal blastomeres of four-cell embryos were each injected with 1 ng of RNAs encoding Dsh-GFP or DsNLSm. Embryonic lysates were collected at stage 10.5 for *in vitro *JNK activity assay using anti-phospho-specific c-Jun antibodies. Total GST-c-Jun levels were assessed with anti-GST antibodies. Dsh-GFP and DsNLSm were equally expressed, as monitored with anti-Dvl2 antibodies; β-tubulin served as a loading control.

Besides the β-catenin pathway, Dsh also functions in a planar cell polarity (PCP) pathway, which involves Rho GTPase and JNK and controls morphogenetic movements in early embryos [8,9,37-39]. We asked whether mutations in DsNLSm influence the β-catenin pathway exclusively or affect the PCP pathway as well. First, we observed that both Dsh-GFP and DsNLSm-GFP were efficiently recruited to the cell membrane by overexpressed Xfz8, a Frizzled family member [40] (Figure 8a). As Dsh relocalization to the cell membrane in response to Frizzled is associated with its ability to signal in the PCP pathway [7,8], this observation suggests that DsNLSm can respond to Frizzled signaling independent of β-catenin.

In *Xenopus*, the PCP pathway involving Dsh is implicated in the control of convergent extension movements [24,41,42]. Overexpression of the Xdd1 deletion mutant leads to the development of short embryos when expressed in dorsal marginal cells ([24]; Figure 8b). Severe convergent extension defects (Figure 8b) were observed in 22%, and mild defects were observed in 28% of the embryos injected with Xdd1 RNA (N = 35). In contrast, only mild morphogenetic defects were observed in embryos coinjected with Dsh (15%; N = 40) or DsNLSm RNA (18%; N = 39), indicating that both Dsh and DsNLSm partially rescued the effect of Xdd1. This indicates that DsNLSm is active in noncanonical PCP-like signaling. We also examined whether DsNLSm activates c-Jun N-terminal kinase (JNK), which is thought to function downstream of Dsh in the PCP pathway [8,37-39]. Both DsNLSm and Dsh activated JNK at equivalent levels (Figure 8c), suggesting that nuclear localization of Dsh is not required for its function in noncanonical signaling.

### Nuclear accumulation of Dsh following Wnt3a stimulation

**Figure 9:**
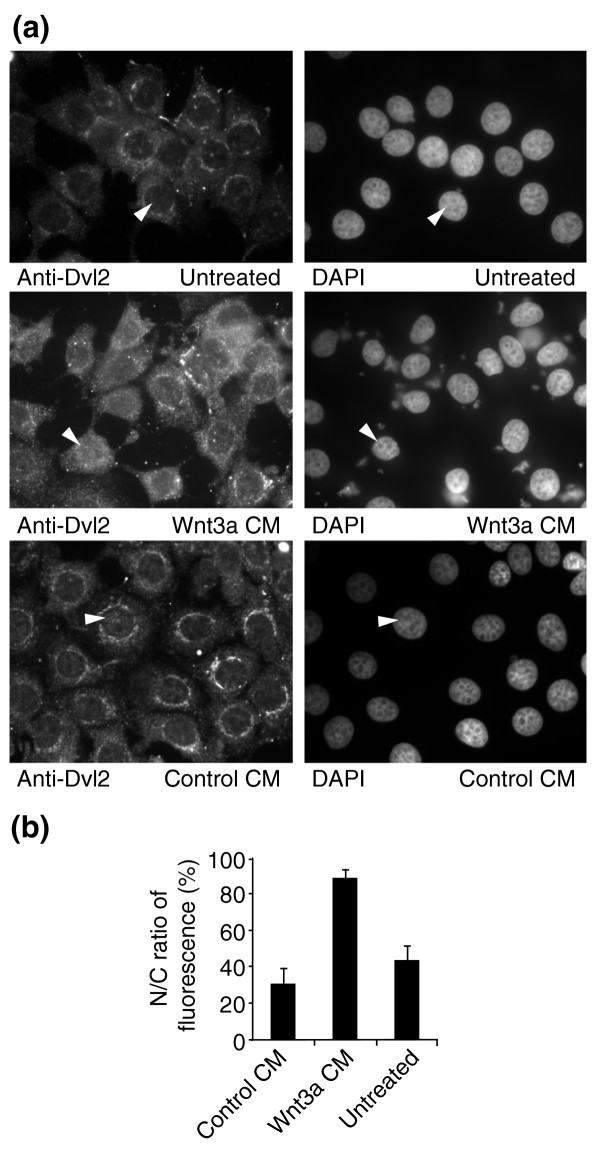
Nuclear translocation of Dvl2 upon Wnt3a treatment. **(a) **MCF7 cells were treated either with Wnt3a-conditioned or control medium for 6 h, fixed and immunostained with anti-Dvl2 antibodies. In control cells, cytoplasmic and perinuclear staining is visible. Wnt3a-conditioned, but not control, medium enhanced nuclear translocation of Dvl2. DAPI staining indicates the position of cell nuclei. Corresponding cells are shown by arrowheads. **(b) **Nuclear/cytoplasmic (N/C) ratios of fluorescence were calculated for each panel in (a) as the mean +/- standard deviation.

Our findings are consistent with a scenario in which Wnt signaling may cause nuclear translocation of Dsh followed by formation of a stable β-catenin/Tcf3 complex and transcriptional activation of target genes. In support of this hypothesis, Dsh was reported to move to the nucleus in response to Wnt signaling in primary embryonic kidney cells [17]. In Rat-1 cells, we did not detect a significant change in Dsh distribution in response to Wnt signals (data not shown), possibly due to highly efficient nuclear export of Dsh in these cells. But immunofluorescence staining for Dvl2 revealed the nuclear accumulation of the protein in HEK293 and MCF7 cells after 3–6 h stimulation with Wnt3a-containing medium (Figure 9a, and data not shown). The effect was quantified by measuring nuclear to cytoplasmic (N/C) ratios of fluorescence intensity. The N/C ratio averaged 28% after 6 h treatment with the control medium, but increased to 91% after stimulation with Wnt3a-conditioned medium (Figure 9b). These observations are consistent with the view that Dsh regulates Wnt-dependent gene targets in the nucleus.

### A role for Dsh in the nucleus

In the current view, Wnt signaling causes inactivation of the β-catenin degradation complex, leading to stabilization and nuclear translocation of β-catenin [3]. Given that Dsh is genetically upstream of the β-catenin degradation complex [3,4] and that β-catenin degradation is thought to occur in the cytoplasm [43], Dsh nuclear import is unexpected. Nevertheless, our data demonstrate that Dsh shuttles between the cytoplasm and the nucleus and that its presence in the nucleus is critical for signaling. One explanation of these results is that β-catenin degradation may occur in the nucleus. Consistent with this possibility, APC, Axin and GSK3, components of the β-catenin degradation complex, have also recently been found to shuttle between the cytoplasm and the nucleus [22,23,44-47]. Moreover, Frat/GBP, a positive regulator of β-catenin, has been reported to expel GSK3 from the nucleus [47]. We show that the ability of Dsh constructs to enter the nucleus correlates with their ability to stabilize β-catenin (Figure 5c). These observations indicate that Wnt/β-catenin signaling may depend on the nuclear localization of pathway components.

Alternatively, nuclear localization of Dsh may affect β-catenin stability indirectly, by regulating protein interactions that sequester β-catenin in the nucleus, thereby preventing its cytoplasmic degradation [48]. Although we did not detect a significant change in nuclear import of β-catenin-GFP in *Xenopus *ectoderm cells overexpressing Dsh (data not shown), this process may be cell-context-dependent. On the other hand, we recently showed that Frodo, a nuclear Dsh-interacting protein, associates with Tcf3 and influences Tcf3-dependent transcription [49]. It is thus possible that Frodo links Tcf3 and Dsh to regulate Wnt target genes. Future studies should examine molecular components critical for the nuclear function of Dsh.

## Materials and methods

### DNA constructs

GFP-tagged Dsh constructs were all derived from the DshGFP-RN3 plasmid that encodes the Xdsh protein fused at amino acid 724 to the first amino acid of GFP (Figures 1a, 4a). Ds1 lacks the first 332 amino-terminal amino acids. Ds2 is the carboxy-terminal deletion of Xdsh, starting with amino acid 383. Ds3 lacks amino acids 334–381. In DsNLSm, the IVLT residues at positions 334–337 were replaced with AVGA, whereas in DsSNLS the same region is replaced with KKKRK, the SV40 T antigen NLS [27]. In DsNESm, L513 and L515 were substituted for alanines.

To generate these constructs, DshGFP-pRN3 was used as a template. The in-frame deletion in Ds3 was made by PCR. Other GFP fusion constructs were synthesized with specific primers and PfuI DNA polymerase followed by DpnI digestion of the template [50]. The following primers were used: 5'-GTCCATAAACCGGGGCCCGCAGTCGGCGCCGTGGCCAAATGCTGG-3' for DsNLSm; 5'-ACACTAGGCCGCAGAATGCCCATTGTCCTGACCGTG-3' for Ds1; 5'-TCCATAAACCGGGGCCAAAGAAGAAGCGAAAGGTGGCCAAATGCTGGGA-3' for DsSNLS; 5'-TTCCCAGTGTACCCCGGGGCCATGGTGAGCAAGGGC-3' for Ds2, and 5'-GAGAACTATGACCAACGCTAGCGCGAATGACAACGATGGAT-3' for DsNESm. All constructs were verified by sequencing. Myc-tagged Dsh mutant constructs were made by replacing mutated regions with corresponding regions of Myc-Dsh [24]. Cloning details are available as an Additional data file with the online version of this article.

### Embryo culture, axis-induction assay and axis-extension assay

*In vitro *fertilization, culture and microinjections of *Xenopus *eggs were essentially as described previously [24]. Stages were determined according to Nieuwkoop and Faber [51]. Axis induction was carried out by injecting mRNAs encoding different Dsh constructs (1 ng) into a single vegetal ventral blastomere at the 4–8-cell stage and assessed when the injected embryos reached stage 36–40. To monitor axis extension defects, 0.6 ng of Xdd1 RNA was injected alone or with 2 ng of Dsh or DsNLSm RNA into two dorsovegetal blastomeres of 4-cell embryos and the injected embryos were allowed to develop until sibling embryos reached stage 32.

### GFP fluorescence and luciferase assay

For subcellular localization of Dsh-GFP constructs, mRNAs were injected into the animal pole region of 2–4-cell embryos. Animal cap explants were dissected at stages 9–10.5, incubated for 60 min in 10 mM N-ethylmaleimide (NEM; Sigma, St Louis USA) in 0.8 × MMR (Marc's Modified Ringer's solution, 1 × MMR: 100 mM NaCl, 2 mM KCl, 1 mM MgCl2, 2 mM CaCl2, 5 mM HEPES, pH 7.4), or in control (0.8 × MMR), then fixed in 4% paraformaldehyde in phosphate-buffered saline (PBS) for 30–45 min, washed three times in PBS, and mounted in 70% glycerol, 30% PBS containing 25 mg/ml of diazabicyclo(2,2,2)-octane (Sigma). Leptomycin B was used at 50 ng/ml in low-calcium medium (76 mM NaCl, 1.4 mM KCl, 0.2 mM CaCl_2_, 0.1 mM MgCl_2_, 0.5 mM Hepes, 1.2 mM sodium phosphate, (pH 7.5), 0.6 mM NaHCO_3 _and 0.06 mM EDTA) for one hour prior to fixation. In some experiments, nuclei were stained by addition of 1 μg/ml 4,6-diamidino-2-phenylindole (DAPI) to the final PBS wash. For membrane localization studies, Xfz8 RNA was coinjected with RNAs encoding the Dsh constructs in the animal-pole region; animal-cap explants were dissected at stage 9–9.5 and mounted for observation. Fluorescence was visualized using a Zeiss Axiophot microscope.

For luciferase assays, pSiaLuc reporter plasmid (20–40 pg) was coinjected with mRNAs encoding Xwnt3a [52] or Xwnt8 [53] and different Dsh constructs into one or two animal-ventral blastomeres or into one ventral-vegetal blastomere at the 4–8-cell stage. Luciferase activity was measured as described [29].

### Tissue culture, immunocytochemistry and subcellular fractionation

Rat-1 fibroblasts, human embryonic kidney (HEK) 293 cells and MCF7 human breast carcinoma cells were cultured in 1 × Dulbecco's Modified Eagle Medium (DMEM; Gibco/Invitro-gen, Carlsbad, USA) supplemented with 10% fetal calf serum and 5 μg/ml gentamicin. Conditioned medium was prepared from L cells stably transfected with Wnt3a as described [54], with the medium from untransfected L cells serving as a control.

For immunocytochemistry, HEK293 cells were treated with 50 ng/ml LMB for 14 h while MCF7 cells were treated with Wnt3a or control conditioned medium for 1, 3, 6 or 8 h. Cells were fixed with 4% paraformaldehyde, immersed in methanol, and incubated with anti-Dvl2 antibodies and then Cy3-conjugated anti-rabbit IgG. Nuclei were stained by addition of 1 μg/ml DAPI as described for animal-cap cells. Fluorescence was observed under the Zeiss Axiophot microscope; 10–15 cells from each group were randomly picked up for measurement of the nuclear and cytoplasmic staining intensity using Image-Gauge software (Fuji Film, Tokyo, Japan).

For subcellular fractionation, confluent cultures of Rat-1 cells were harvested by scraping plates and resuspended in hypotonic lysis buffer containing 1 mM EGTA, 1 mM EDTA, 2 mM MgCl_2_, 10 mM KCl, 1 mM DTT, 10 mM β-glycerophosphate, 1 mM sodium orthovanadate, 1 μg/ml leupeptin, 1 μg/ml aprotinin, and 1 μg/ml pepstatin. Cells were swollen for 30 min, and broken open with 25 strokes in a tight fitting Dounce homogenizer. Lysates were layered into tubes containing 1 M sucrose in hypotonic lysis buffer, and spun at 1600 × *g *for 10 min. Supernatant remaining above the sucrose cushion was used as the cytoplasmic fraction. The pellet, containing nuclei, was resuspended in an equivalent volume of hypotonic lysis buffer.

### Immunoprecipitation and western blotting

Immunoprecipitation and western analysis were carried out with cell and embryo lysates as described [14]. To prepare embryo lysates at stage 10+, four animal blastomeres of 4–8-cell embryos were injected with RNAs encoding different forms of Dsh, ΔRGS-Axin [32], Flag-β-catenin [55], CK1ε [30] and HA-XARP [20]. To generate anti-Xdsh polyclonal antibodies, rabbits were immunized with a carboxy-terminal half of Xdsh (amino acids 301–736) fused to GST. First, GST beads were used for purification of anti-GST antibodies. Subsequently anti-Xdsh antibodies were affinity-purified on GST-Xdsh (301–736) beads. Polyclonal anti-Dvl2 antibody was generated in rabbits and affinity-purified on PVDF membrane blotted with human Dvl2 (79–249) [56]. A small aliquot of anti-human Dvl2 was obtained from M. Snyder (Yale University, New Haven, USA). Anti-GAPDH antibody was a gift from A. Stuart-Tilley and S. Alper (Beth Israel Deaconess Medical Center, Boston, USA), anti-lamin antibody was from F. McKeon (Harvard Medical School, Boston, USA). Anti-β-tubulin antibodies were from BioGenex (San Ramon, USA), anti-Flag M2 antibody was from Sigma and anti-CK1ε antibodies were from BD Biosciences (Palo Alto, USA). Anti-Myc and anti-HA monoclonal antibodies are hybridoma supernatants of 9E10 and 12CA5 cells (Roche Applied Science, Indianapolis, USA).

### JNK assay

Four-cell embryos were injected with 4 ng Dsh or DsNLSm RNA into four animal blastomeres. Embryo lysates were prepared at stage 10.5 and *in vitro *kinase assays were carried out essentially as described [57], except that phosphorylated c-Jun-GST was detected with anti-phospho-c-Jun-specific antibodies (Cell Signaling Technology, Beverly, USA) by western blotting rather than with autoradiography.

## Additional data files

The following is provided as an additional data file with the online version of this article. Additional data file 1, containing cloning details of Dsh mutant constructs.

## Supplementary Material

Additional data file 1 Cloning details of Dsh mutant constructs Click here for additional data file

## Figures and Tables

**Table 1:** Axis induction by Dsh constructs

Injected RNA	Total number of injected embryos	Complete secondary axes (%)	Partial secondary axes (%)
Experiment 1			
Dsh-GFP	150	46.6	25.3
DsNESm-GFP	194	54.6	30.4
			
Experiment 2			
Dsh-GFP	144	28.5	45.1
DsNLSm-GFP	149	0.7	39.5
DsSNLS-GFP	137	24.0	42.3

Embryos were injected as described in Figure 1e,f. Partial secondary axes are defined by a morphologically visible ectopic neural tube up to the hindbrain level. Complete axes are defined by the presence of the secondary head structures, including eyes and cement glands. The frequency of secondary axes in uninjected embryos was less than 1%. Data pooled from several independent experiments are shown.
