# Supplementary material for: Nuclear localization is required for Dishevelled function in Wnt/β-catenin signaling
Source: J Biol. 2005 Feb 15;4(1):3. doi: 10.1186/jbiol20 (PMC551520; doi:10.1186/jbiol20)
Supplement: Additional data file 1 — Cloning details of Dsh mutant constructs [file jbiol20-s1.pdf]

Additional data file

DNA constructs (cloning details)

The DshGFP-RN3 plasmid was constructed by ligating *Hind*III-*Nco*I fragment of Xdsh-pSP64R1 [58], *Nco*I-*Not*I fragment of pEGFP-N1 (Clonetech, Palo Alto, USA) and *Hind*III-*Nco*I digested pBluescript-RN3 [59]. Ds3 was generated by PCR with the primer 5′-AAGGGCCCAGCGCCT-CCATG-3′ and the standard T7 primer on a template corresponding to Xdsh-pBluescript-SK [58] with internal *Xho*I deletion. The PCR fragment was digested with *Apa*I and *Xho*I and subcloned into *Apa*I-*Xho*I-digested DshGFP-pRN3. To generate Myc-Ds3, Myc-DsNLSm and Myc-DsSNLS, *Pst*I-*Xho*I fragments from Ds3, DsNLSm and DsSNLS were inserted into *Pst*I-*Xho*I digested Myc-Xdsh, respectively [24].

Ds1, Ds2, DsNLSm, DsSNLS and DsNESm were generated by ‘one-primer’ *Pfu*I-mediated mutagenesis modified from Makarova *et al.* [50], using the DshGFP-pRN3 template. A reaction contained 300 ng of template DNA (DshGFP-pRN3), 5 picomoles of a specific primer, 5 nanomoles of each dNTP and 1.25 U of Pfu Turbo DNA polymerase (Stratagene, Cedar Creek, USA) with the corresponding enzyme buffer in a total volume of 25 μl. DNA was denatured at 95 °C for 3 min following by 18 cycles of (95 °C, 15 s; annealing temperature, 1 min; 68 °C, 12 min). The annealing temperature varied from 45 °C to 65 °C, depending on the primary sequence of the primer; 10 μl of the reaction were digested with 1 μl of *Dpn*I for 2 h at 37 °C to digest methylated template DNA and transformed into TG1 competent bacteria. Colonies containing mutations were identified by restriction enzyme digestion, as each primer was designed to create an additional enzyme site. Mutagenesis was confirmed by sequencing.
